# An update on clinical significance of use of THSD7A in diagnosing idiopathic membranous nephropathy: a systematic review and meta-analysis of THSD7A in IMN

**DOI:** 10.1080/0886022X.2018.1456457

**Published:** 2018-04-06

**Authors:** Song Ren, Changwei Wu, Yuan Zhang, Amanda Y. Wang, Guisen Li, Li Wang, Daqing Hong

**Affiliations:** a Renal Department and Nephrology Institute, Sichuan Provincial People’s Hospital, School of Medicine, University of Electronic Science and Technology of China, Chengdu, China;; b The George Institute for Global Health, University of Sydney, Sydney, Australia

**Keywords:** Thrombospondin type 1 domain–containing 7A, idiopathic membranous nephropathy, meta-analysis, systematic review, diagnostic value

## Abstract

**Background**: THSD7A is a new target antigen of idiopathic membranous nephropathy (IMN). Moreover, malignancies are also found in patients with THSD7A-positive membranous nephropathy. We aimed to systematically evaluate the prevalence of THSD7A in IMN patients and malignancies in THSD7A-positive patients.

**Methods**: We searched English and Chinese database to 31 December 2017 with the term ‘THSD7A’ or ‘thrombospondin type 1 domain-containing 7A’. Meta-analysis was used to explore the positive rate of THSD7A in the IMN patients. Subgroup analysis was performed according to the race, sample size, and detecting method of THSD7A.

**Results**: Ten studies involving 4121 participants were eventually included. The prevalence of THSD7A was 3% (95% CI, 3%–4%) in all patients and 10% (95% CI, 6%–15%) in PLA2R-negative patients. 77 patients had positive circulating antibodies, and the prevalence of THSD7A was also low at 3% (95% CI, 2%–4%). Overall, 72 patients had positive THSD7A staining on renal biopsy, and the prevalence was 3% (95% CI 2%–4%). Subgroup analysis showed significant differences in the prevalence of THSD7A based on the study sample sizes, however, no significant differences were seen in different ethnic groups. Furthermore, among THSD7A-positive patients, 3/10 studies reported malignancies with the incidence varied from 6% to 25%.

**Conclusions**: The prevalence of THSD7A is more common in the PLA2R-negative patients than the IMN patients. Screening for malignancies in THSD7A-positive MN patients is recommended.

## Introduction

Idiopathic membranous nephropathy (IMN) is the most common cause of primary nephrotic syndrome in adults. About one-third of patient progress to end-stage kidney disease. The diagnosis of membranous nephropathy is based on renal biopsy, characterized as deposition of immune complex along the glomerular basement membrane [1]. In recent years, the incidence of IMN showed a significantly increasing trend. Beck et al. [2] identified a circulating autoantibody reactive to the transmembrane glycoprotein M-type phospholipase A2 receptor 1 (PLA2R) on the human podocyte. PLA2R is the first antigenic target recognized in IMN in adults.

Thrombospondin type 1 domain–containing 7A (THSD7A) was described as a second autoantigen involved in adult IMN. PLA2R and THSD7A are two targets that are assumed to cause IMN in the majority of patients. The prevalence of THSD7A is reported ranging from 1 to 10% based on different studies. For example, Tomas et al. [3] found that about 10% patients with IMN had circulating autoantibodies to THSD7A but not to PLA2R, THSD7A maintained positive staining in idiopathic membranous glomerulopathy by immunofluorescence and immunohistochemical analyses but not in other glomerular diseases or healthy controls. Iwakura et al. [4] found that enhanced granular expression of THSD7A was detected in 9.1% Japanese patients with idiopathic MN. Hoxha et al. [5] screened for the presence of THSD7A by assessing the serum samples of 1276 patients with MN from three different cohorts. Among them, 40 patients with THSD7A-associated MN were identified, and eight of them developed a malignancy within a median follow up of 3 months from diagnosis of MN. This result suggested that THSD7A-associated MN may be associated with malignancy.

In order to better understand the role of THSD7A in MN (with or without malignancies), we performed a systematic review and meta-analysis to explore the prevalence of THSD7A in MN and the prevalence of malignancies in THSD7A-positive patients.

## Subjects and methods

### Data sources and searches

We searched MEDLINE via Ovid (from 1946 through Dec 2017), Embase via OVID (from 1980 through Dec 2017), Cochrane Library database via OVID (Cochrane Central Register of Controlled Trials; up to Dec 2017) with the term ‘THSD7A’ or ‘thrombospondin type 1 domain-containing 7A’, without language restriction to identify relevant studies. We also carried out hand searches of bibliographies, Internet searches of unpublished studies in the form of posters or abstracts.

### Study selection

The review of eligible scientific reports, data extraction, and quality assessment was conducted independently using a standardized approach (SR and DH). Any disagreement was adjudicated by a third reviewer (LG). Studies pertaining to the determination of THSD7A in patients with MN were included for analyses if the following criteria were met: 1) they were studies of patients with MN including cross-sectional, prospective, and retrospective studies; 2) data on THSD7A prevalence were demonstrated. Studies with less than 50 participants were excluded. Duplicate reports, case reports, and studies that did not report one or more of the study outcomes of primary interest (prevalence of serum THSD7A antibody or prevalence of renal tissue THSD7A antibody) were excluded.

### Data extraction and quality assessment

The quality of each study was independently evaluated by each investigator using the Newcastle-Ottawa quality assessment scale. Discrepancies in data extraction and quality assessment were resolved by the third reviewer. The following variables were extracted: author’s name(s), publication year, country where the study was conducted, period of follow-up, number of patients studied, number of THSD7A-positive patients, patients’ age, and gender.

### Outcomes

Clinical outcomes included prevalence of positive THSD7A antibody in all MN patients as well as in PLA2R antibody negative patients, defined as the prevalence of positive serum THSD7A antibody, prevalence of positive THSD7A in tissue biopsy, and the prevalence of composite of serum or THSD7A antibody positivity.

### Data synthesis and analysis

Prevalence of THSD7A antibody positivity was calculated (and 95% confidential intervals) for each study. Summary estimates were obtained using a fixed effects model. The percentage of variability across the pooled estimates attributable to heterogeneity beyond chances was estimated using the *I*^2^ statistic, and the *p* values for heterogeneity were calculated [6]. An *I*^2^ statistic of 0–25% was considered to reflect a low likelihood, 26–75% a moderate likelihood and 76–100% a high likelihood of differences beyond chances. A *p* value of <.05 represented for heterogeneity. A 95% confidence interval that did not span unity indicated a statistically significant result for an outcome. Statistical analyses were performed using Revman 5.3 software. Subgroup analyses according to race and sample sizes were performed. Malignancies in THSD7A positive patients were only described but not analyzed due to the limited numbers of reports.

## Results

### Description of the included studies

**Figure 1.**
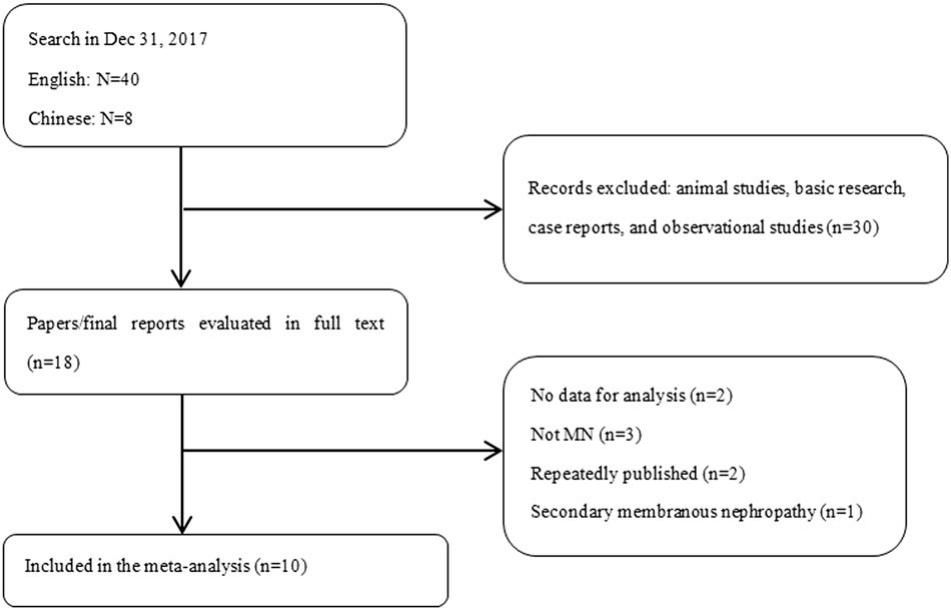
Study selection flow chart.

The search retrieved 71 citations for screening, from which 10 studies [3–5,7–13] involving 4121 participants were eventually included (Figure 1). Seven studies [3–5,7,9,10,12] were published in English literature, and three studies [8,11,13] were published in Chinese literature. Two studies [12,13] were only published in the form of an abstract. Three studies [3,7,11] found that THSD7A-associated MN may be associated with tumors. The study selection was summarized in Table 1.

**Table 1.** The positive THSD7A in including studies.

		THSD7A(+)		THSD7A(–)		
Study	Specimens	PLA2R(+)	PLA2R(−)	PLA2R(+)	PLA2R(−)	Total
Tomas 2014	IMN(Serum)	0	15	329	139	483
Iwakura 2015	IMN(Tissue)	0	5	29	21	55
Sharma 2017	IMN(Tissue)	2	29	–	–	1318
Hayashi2016	IMN(Tissue)	1	2	36	20	59
Hoxha 2016	IMN(Serum)	0	39	531	706	1276
	IMN(Tissue)	0	37	531	708	1276
Li Jianing 2015	IMN(Serum)	1	3	30	94	128
	MN(Tissue)	1	3	30	94	128
Han Didi 2016	IMN(Tissue)	0	4	46	13	63
Wang Jia 2017	IMN(Serum)	2	6	392	178	578
	IMN(Tissue)	2	6	–	–	578
Wen Liyin 2016	IMN(Tissue)	–	2	76	–	86
Dahan 2017	IMN(Serum)	–	2	55	–	75

### THSD7A prevalence

**Figure 2.**
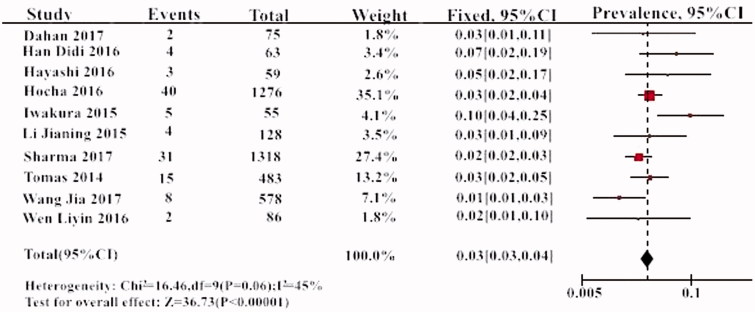
The prevalence of THSD7A in all studies.

**Figure 3.**
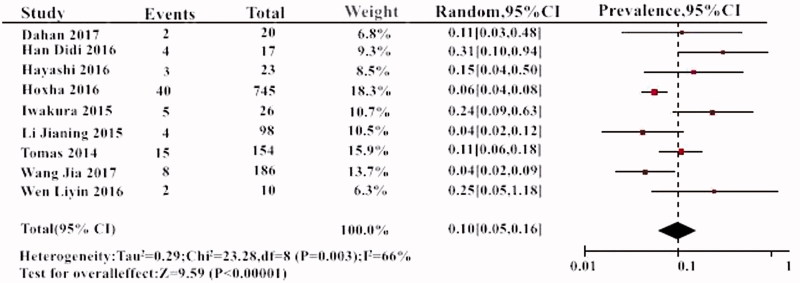
The prevalence of THSD7A in PLA2R-negtive patients.

The prevalence of THSD7A within the 10 individual study populations ranged from 1 to 10%, with an overall meta-analytical prevalence of 3% (95% CI, 3–4%). A low level of heterogeneity was noted (*I*^2^= 45%, *p* > .05) (Figure 2). The prevalence of THSD7A in PLA2R-negative patients was 10% (95% CI, 5–16%) (Figure 3).

**Figure 4.**
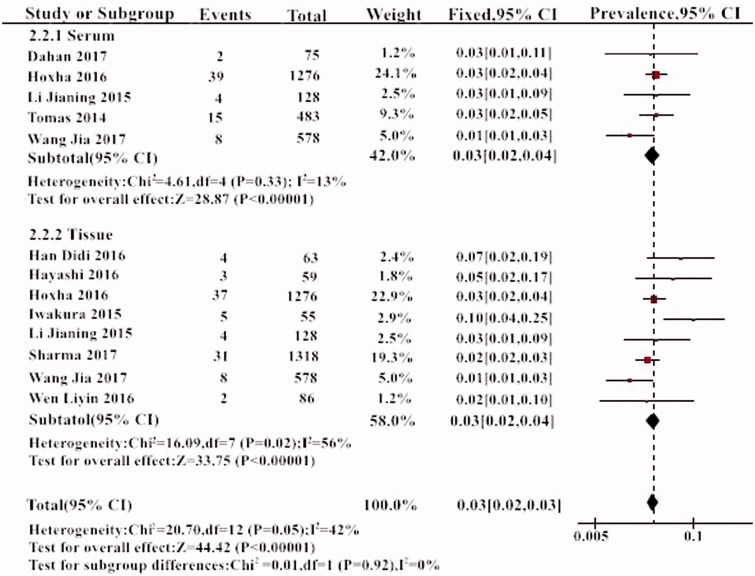
The results of subgroup analysis of the prevalence of THSD7A in different detection method.

Subgroup analysis was performed according to the way of detecting THSD7A. Among a total of 2626 patients who were tested for circulating THSD7A antibodies, 68 patients were positive for THSD7A. The positive serum THSD7A was 3% (95% CI, 2–4%). The positive THSD7A antigen deposition in renal tissue by immunohistochemistry was found in 94 of 3563 patients. The prevalence of positive THSD7A in renal tissue was 3% (95% CI, 2–3%). There was no statistically significant difference between these two methods. (*p* > .05) (Figure 4).

**Figure 5.**
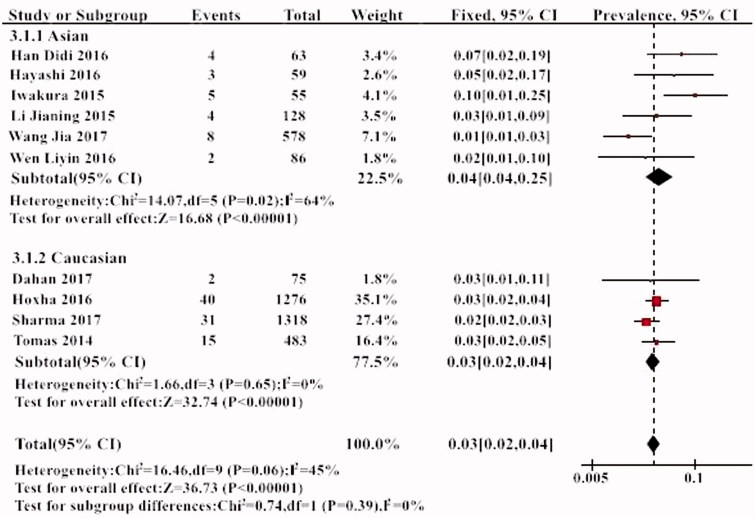
The results of subgroup analysis of the prevalence of THSD7A in different races.

**Figure 6.**
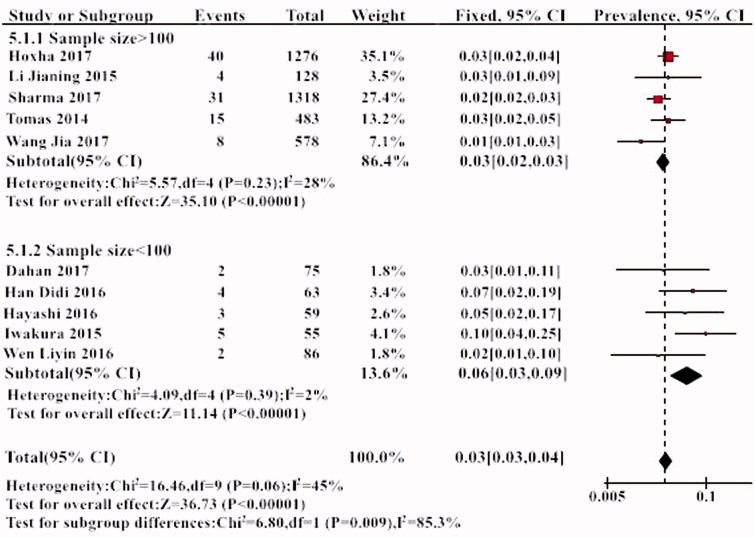
The results of subgroup analysis of the prevalence of THSD7A in different sample size.

Further subgroup analysis was performed according to the different ethnic groups. THSD7A positivity was 3% in the Caucasian and 4% in the Asian population. However, this difference was not statistically significant (Figure 5). Further subgroup analysis also found that the incidence of positive THSD7A in the studies with small sample sizes was higher (6%) than those with large sample size (*p* < .05) (Figure 6).

The positive rate of THSD7A was very low in secondary membranous nephropathy (SMN). Among the studies included in our review, a total of 157 SMN patients were detected for THSD7A. At last, only two patients with SMN were found to have positive serum THSD7A antibody. In the European cohort, a 29-year-old female patient was diagnosed with systemic lupus erythematosus had positive serum THSD7A antibody, and in the Boston cohort, a 77 years old male SMN patient had active prostate cancer at the time of the diagnosis of MN. In another study [14], the authors analyzed 24 patients with psoriasis related MN and did not find positive THSD7A in the serum.

### Incidence of malignancy in THSD7A-associated MN

Three studies found that THSD7A-associated MN may be associated with tumors. A total of 9 patients with positive THSD7A had malignancies reported. A Chinese study [11] found that one patient with kidney cancer appeared positive for both PLA2R and THSD7A in the tissue staining. Another study [5] found that 8 of 40 patients developed a malignancy within a median time of 3 months from diagnosis of MN. Tumors were mainly involved in the gastrointestinal and genitourinary system. Finally, one study [7] found that among two of 31 patients who had a history of cancer, none of them were diagnosed with malignancy on the follow-up.

### Reporting bias

**Figure 7.**
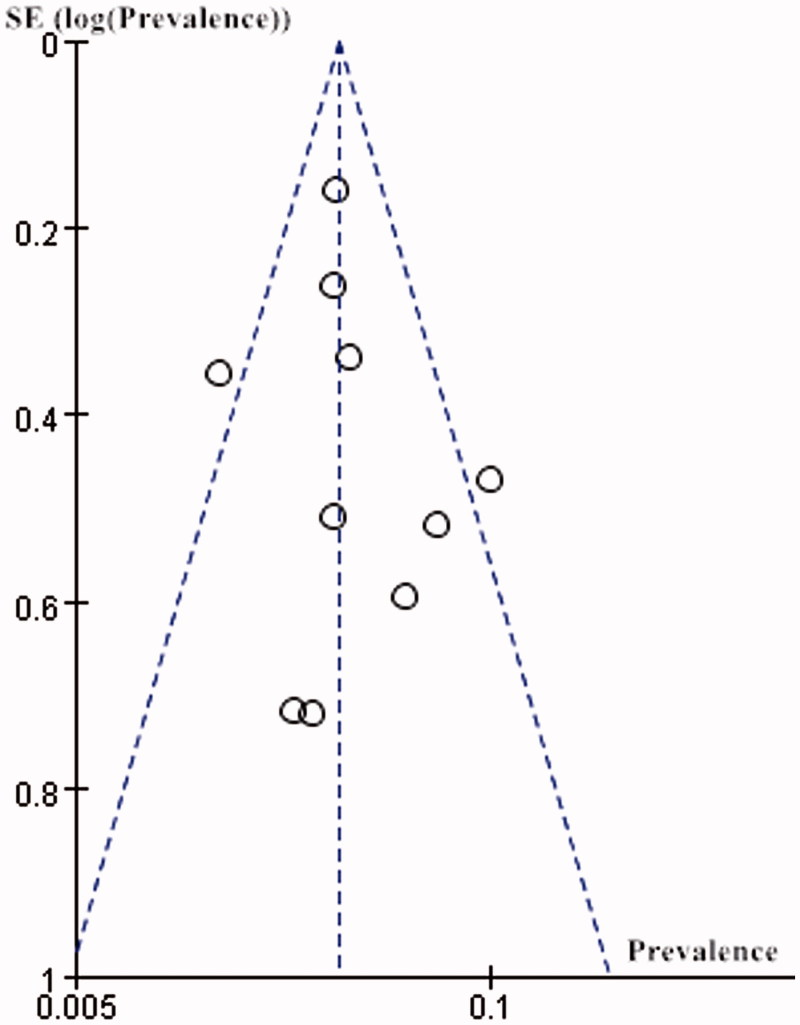
Funnel plot of including studies.

A funnel plot was drawn to describe the report bias of the including studies. The funnel plot was not very asymmetrical, and the bias of the studies was large. However, due to the limited numbers of studies, the power of the test appeared to be too low to distinguish the chance from real asymmetry (Figure 7).

## Discussion

IMN is the most common cause of nephrotic syndrome in adults. It is an antibody-mediated autoimmune disease, which has been supported by the discovery of neural endopepidase (NEP) and PLA2R. Our analysis demonstrated that the estimated prevalence of THSD7A in patients with IMN was 3% and higher prevalence in the PLA2R-negtive patients was seen at 10%. There were no significant differences between different detection methods and different races.

In 2009, Beck et al. [2] first reported that M-type PLA2R was a major target antigen for IMN and about 70% of patients with IMN had autoantibodies to PLA2R in their serum. This biomarker then serves not only as a maker of diagnosis of IMN but also an indicator for the disease activity. THSD7A, firstly reported in 2014, was recognized as a new marker and assumed to play a similar role to PLA2R in IMN. Tomas et al. [3] found 15 of 154 patients with IMN had circulating autoantibodies to THSD7A but not to PLA2R. Subsequently, more clinical studies have found positive THSD7A was detected in patients with IMN, either by testing for positive circulating antibodies in the serum or THSD7A antigen deposition in renal tissue by immunohistochemistry. These findings have confirmed that THSD7A is as a maker of diagnosing IMN.

THSD7A is a soluble form of membrane-associated N-glycoprotein [15]. Initially, it expressed in the placenta vasculature and in human umbilical vein endothelial cells. As an endothelial protein, THSD7A mediates tube formation and endothelial cell migration [16]. Like PLA2R1, THSD7A has an extracellular region which is consisted of disulfide-bonded and N-glycosylated domains. The pathogenesis of THSD7A-associated MN may be related to the immune complex formation with podocyte-associated THSD7A. Nicola et al. [17] transferred anti-THSD7A antibodies from 2 patients with THSD7A-associated MN in mice. The results demonstrated that human anti-THSD7A antibodies can bind to the murine antigen both *in vivo* and *in vitro* studies. Moreover, the administration of both anti-THSD7A antibody-containing serum and isolated THSD7A-specific IgG to mice led to the development of proteinuria and a histo-morphological pattern of MN. They also found that THSD7A-expressing glomerular epithelial cells underwent marked cytoskeletal rearrangement, with an alteration in cellular morphology and focal adhesions upon binding of the antibody to the cell membrane. These findings indicated that anti-THSD7A antibodies might directly interfere with podocyte integrity, leading to cell damage and proteinuria.

Sharma et al. [7] detected the serum samples from 24 patients whose tissue had positive staining for THSD7A, and found all of them were positive for THSD7A antibodies. While the serum antibodies were negative in 20 patients who had no THSD7A stain in tissues. This indicates the positive rate of THSD7A of serum and renal tissue was similar. THSD7A is a new antigen, how the antibody is generated is unknown now, our study showed a similar positive rate of this antibody in serum an tissue, but could not draw any conclusion, this phenomenon needs further studies.

The diagnosis of THSD7A-related MN can be performed by detection of antibodies in the serum and immunohistochemistry of renal tissue. Majority of patients with IMN demonstrated positive results in both methods. However, small number of patients only had positive serum antibody or positive deposition of THSD7A in their renal tissues. Therefore, simultaneous detection of antibodies in both serum and renal tissue can increase the sensitivity of the diagnosis, although our results showed there was no significant difference in the disease prevalence between these two detection methods.

Compared with the PLA2R-MN group, the THSD7A-MN group did not show a significant difference in sex, urinary protein excretion, serum albumin, and creatinine levels in the Iwakura’s cohorts [4]. Similarly, there was no significant correlation between the THSD7A-Ab levels and the clinical responsiveness such as the amount of proteinuria. There was no significant association between remission of proteinuria and the THSD7A-Ab levels [5] in IMN patients according to the Hoxha’s study cohort. Furthermore, a study found that patients with THSD7A-associated MN were prone to disease recurrence after renal transplantation. Nicola et al. [17] described the clinical progress of a male patient with THSD7A-associated MN. After progressing to end stage renal disease (ESRD), he underwent renal transplantation. However, MN rapidly recurred after the transplantation. Enhanced staining for THSD7A was observed in the kidney allograft, and detectable anti-THSD7A antibodies were present in the serum both before and after transplantation, suggesting antibodies induced recurrence of MN in the renal transplant. However, we were unable to prove this hypothesis in our study due to limited available data.

Recent studies have found that THSD7A is closely related to the occurrence of malignancy. In our review, two studies reported malignancies, ranging from 20% to 25% in the THSD7A-positive patients. An increasing number of literatures have revealed that THSD7A-MN patients appear to have an increased risk of development of malignancy. After treatment with immunosuppressives or resection of the tumors for THSDTA-Ab-positive patients, a majority of patients had a significant reduction in the amount of proteinuria. In addition, THSD7A protein was also found in the tumor tissue [5]. THSD7A protein was also detected in follicular dendritic cells in the light zone of the germinal center of the tumor–infiltrated lymph nodes. Immunohistochemical evaluation of the tumor tissue found an increase in THSD7A protein expression, indicating that the tumor was actively synthesizing THSD7A [5]. Hoxha et al. [18] found the messenger RNA for THSD7A was detectable in the gallbladder carcinoma but not in the normal tissue of the gallbladder. After investigating the role of THSD7A as a new potential tumor antigen by evaluating over 20,000 tissue spots in more than 70 different tumor entities by immunohistochemistry using tissue microarrays, the study found that THSD7A expression was highly variable in different neoplasia [18]. Although the causal relationship between malignancy and THSDTA-Ab-positive MN remains unclear, screening, and monitor malignancies in THSD7A-Ab-positive MN needs to be considered, especially for gastrointestinal and genitourinary cancer.

To our knowledge, this is the first study that systematically assessed the prevalence of positive THSD7A in IMN patients and explored potential relationship of positive THSD7A and malignancies. However, our review also has some limitation. Firstly, the studies involved in our review had relatively small sample size, which has reduced the statistical power. Secondly, the detailed information on clinical characteristics in studies published in the abstract form was not complete. Thirdly, the potential for reporting bias exists, which may have an impact on the results.

## Conclusions

The prevalence of THSD7A is not uncommon in the patients with IMN, and more common in the PLA2R-negative patients. Malignancies should be screened and closely monitored in THSD7A-positive membranous nephropathy patients.

## Abbreviations

IMN: idiopathic membranous nephropathy
SMN: secondary membranous nephropathy
THSD7A: thrombospondin type 1 domain–containing 7 A
PLA2R: phospholipase A2 receptor
IFT: indirect immunofluorescence test
NEP: neural endopeptidase
ESRD: end stage renal disease
